# Formal Comment to Pettengill: The Time to Most Recent Common Ancestor Does Not (Usually) Approximate the Date of Divergence

**DOI:** 10.1371/journal.pone.0134435

**Published:** 2015-08-14

**Authors:** Mark Achtman, Zhemin Zhou, Xavier Didelot

**Affiliations:** 1 Warwick Medical School, University of Warwick, Coventry, CV4 7AL, United Kingdom; 2 Department of Infectious Disease Epidemiology, Imperial College London, London W2 1PG, United Kingdom; Field Museum of Natural History, UNITED STATES

## Abstract

In 2013 Zhou *et al*. concluded that *Salmonella enterica* serovar Agona represents a genetically monomorphic lineage of recent ancestry, whose most recent common ancestor existed in 1932, or earlier. The Abstract stated ‘Agona consists of three lineages with minimal mutational diversity: only 846 single nucleotide polymorphisms (SNPs) have accumulated in the non-repetitive, core genome since Agona evolved in 1932 and subsequently underwent a major population expansion in the 1960s.’ These conclusions have now been criticized by Pettengill, who claims that the evolutionary models used to date Agona may not have been appropriate, the dating estimates were inaccurate, and the age of emergence of Agona should have been qualified by an upper limit reflecting the date of its divergence from an outgroup, serovar Soerenga. We dispute these claims. Firstly, Pettengill’s analysis of Agona is not justifiable on technical grounds. Secondly, an upper limit for divergence from an outgroup would only be meaningful if the outgroup were closely related to Agona, but close relatives of Agona are yet to be identified. Thirdly, it is not possible to reliably date the time of divergence between Agona and Soerenga. We conclude that Pettengill’s criticism is comparable to a tempest in a teapot.

## Introduction

### 1. Population structure of *Salmonella enterica* subspecies *enterica* according to MultiLocus Sequence Typing (MLST)

**Fig 1:**
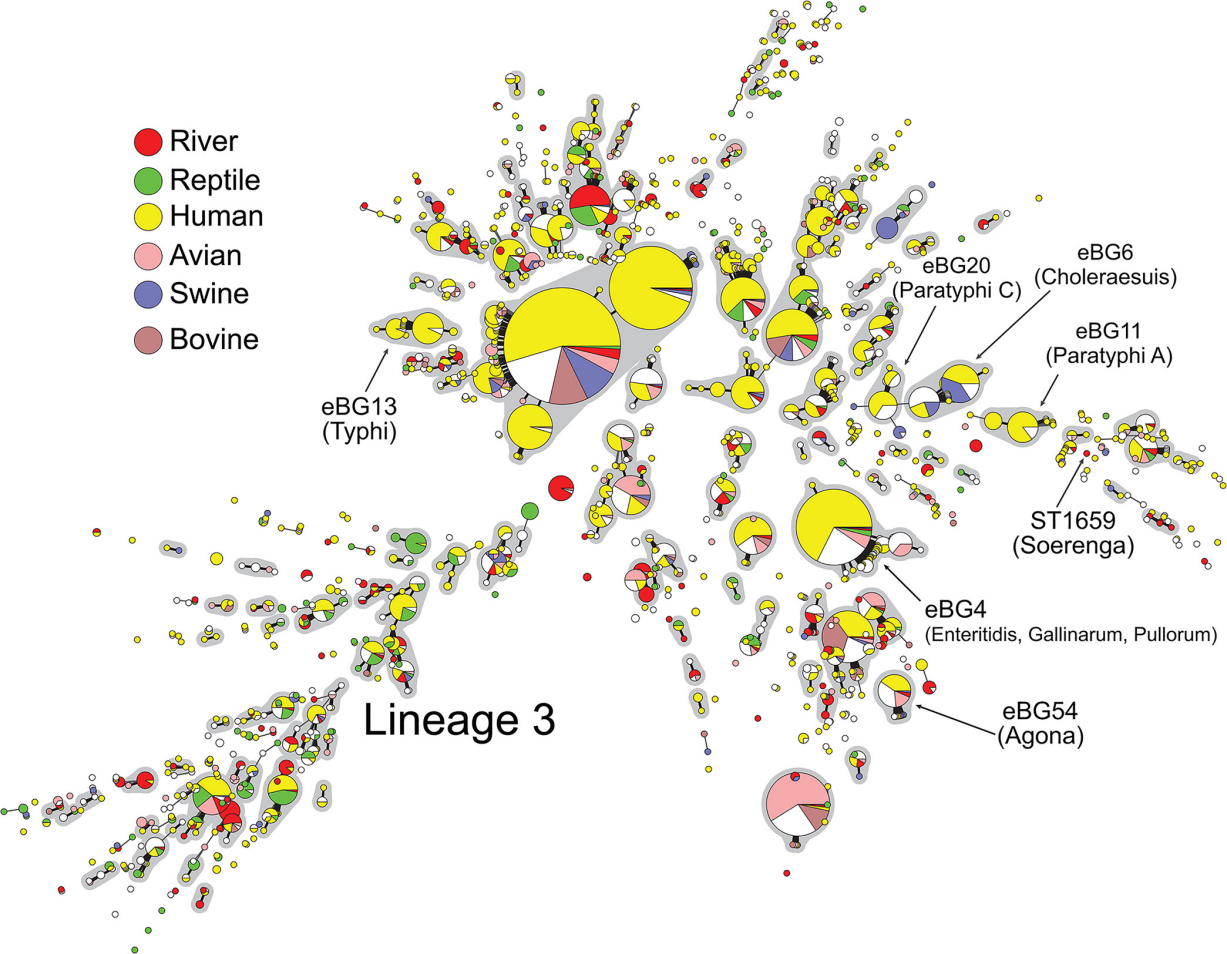
Minimal spanning tree of 150 eBGs and 1,368 STs within 6,309 isolates of *S*. *enterica* subspecies *enterica*. Each circle is one ST, whose radius is proportional to the number of entries of that ST at the *S*. *enterica* MLST website (http://mlst.warwick.ac.uk/, May, 2015), and presented as a pie-chart colored according to source of isolates, or white for isolates from other sources or with missing data. STs that differ by 1/7 MLST loci are connected by a thick line and STs that differ by 2/7 are connected by a thin line. eBGs (groups of STs linked by thick lines) are emphasized by gray shading outside the circles. eBGs and STs referred to explicitly in the Introduction are designated by arrows plus information about their eBG/ST designation and serovar. Lineage 3 is the set of STs and eBGs radiating towards 08:00.

Subspecies *enterica* is commonly isolated from the aqueous environment, but it also causes gastroenteritis and invasive disease in various mammals [1–3]. Medical microbiologists have traditionally assigned serovar designations based on serological reactivity (e.g. Typhi, Paratyphi A, Typhimurium, Enteritidis, Agona, Soerenga) to distinctive groups of these Gram-negative bacteria. More recently, serotyping has been replaced by sequencing seven fragments of housekeeping genes (MLST) [4]. Population genetic analyses of MLST data from 6,309 isolates in 616 serovars of *S*. *enterica* subsp. *enterica* identified 150 discrete, monophyletic and genetically monomorphic clades of sequence types (STs), referred to as eBurst Groups (eBGs) [4] (Fig 1). Some serovars correspond to a single eBG, whereas others do not, and instead reflect phenotypic convergence of unrelated eBGs which express the same serological properties due to their exchange of genes by homologous recombination. In addition, recombination and mutation have resulted in the existence of multiple serovars within some eBGs, such as eBG4 which includes members of serovars Enteritidis, Gallinarum and Pullorum [4]. However, in general, most isolates within an eBG or an individual ST belong to the same or closely related serovars, which explains why serological typing often corresponds to discrete genetic populations.

The genetic relationships between eBGs have not yet been definitively elucidated. Most eBGs have no close relatives according to MLST [4]. Exceptionally, several eBGs show close genetic relationships to others e.g. eBG6 (serovar Choleraesuis) and eBG20 (Paratyphi C). Furthermore, a broad subset of eBGs that are preferentially isolated from the environment or from reptiles form a network that is linked by frequent homologous recombination [5]. This subset was originally designated as clade B [6] but is now referred to as lineage 3 [4,5].

### 2. Genomic studies

MLST has limited resolution because of the small fraction of the genome that is captured when sequencing only 7 gene fragments. However, many of the insights revealed by MLST (Fig 1) have now been substantiated by genomic analyses, which have also provided greater details. Extensive genomic analyses have shown that eBG13 (Typhi) [7], eBG11 (Paratyphi A) [8] and eBG54 (Agona) [9] each corresponds to a distinct, genetically monomorphic clade, as does eBG4 (Enteritidis, Gallinarum, Pullorum) [10]. These conclusions are based on genealogies reconstructed from single nucleotide polymorphisms (SNPs) that mark intra-clade vertical descent, after excluding other, clustered SNPs that were acquired by homologous recombination from unrelated *S*. *enterica*, or are associated with repetitive DNA. For example, in the genealogical tree of eBG54 (Agona) recent recombination with unrelated *S*. *enterica* has imported 42 regions (360 kb) containing 3,164 clustered SNPs at 5/143 nodes *versus* only 846 non-homoplastic, mutational SNPs in the rest of the 4.2 MB core genome [9]. In this case, recombination from external sources resulted in greater genomic divergence, but recombination can also have a convergent effect. For example, homologous recombination between the ancestors of eBG13 (Typhi) and eBG11 (Paratyphi A) resulted in an average nucleotide divergence of 0.2% over one quarter of their genomes in contrast with an average divergence of 1.2% over the remaining three quarters [8,11]. Thus, recombination events can falsify genealogies if they are not accounted for in phylogenetic reconstructions.

Other insights from MLST are also confirmed by sequence analyses of large numbers of gene fragments [5,12] as well as of SNPs from whole genomes [13,14], such as a close relationship between Choleraesuis and Paratyphi C, or between Enteritidis, Gallinarum and Pullorum. The existence of the ancient lineage 3 is also confirmed, manifesting as a distinct branch in phylogenetic trees and a distinct population in population genetic analyses, possibly due to homogenization by frequent homologous recombination. However, these four studies differ dramatically in their conclusions about deep branch structures other than lineage 3, and in the serovars that are assigned to those deeper lineages. These discrepancies may reflect the fact that only few isolates and serovars were included in each study; none cover more than a small fraction of the geographical, temporal and serovar diversity that is provided by the MLST data. Furthermore, the individual studies are difficult to compare because except for Didelot *et al*. [5], they only provide serovar designations, which correlate only imperfectly with eBGs or STs.

### 3. Problems with the choice of serovar Soerenga as a suitable outgroup

Pettengill [15] refers to the distances and topologies calculated by one of these analyses, that of Timme *et al*. [14], which encompassed 156 isolates from 78 of the 2,500 serovars in *S*. *enterica*. The phylogeny reconstructed by Timme *et al*. shows three Agona isolates clustered tightly together, which belong to MLST STs 13 and 1215 within eBG54 according to our reanalysis of the raw data, and confirm an association of Agona with eBG54. Timme *et al*. concluded that serovar Agona was polyphyletic because their tree also included one additional Agona isolate (strain 632182–2), which was distantly related to the other three. Our reanalysis of that genome indicates that this exceptional isolate it is a member of ST413 within eBG62, which otherwise contains 26 strains of serovar Mbandaka according to the MLST database (http://mlst.warwick.ac.uk). Agona is normally monophasic, and does not express the *fljB* phase 2 flagellar antigen. However, the genome of strain 632182–2 possesses an intact *fljB* gene, suggesting that it is diphasic. (The sole diphasic Agona that was previously tested by MLST was also not in eBG54.) Thus, this example provides additional support for using MLST-based eBG assignments to interpret genomic sequences of *S*. *enterica*.

Another feature of the Timme *et al*. [14] phylogeny referred to by Pettengill [15] was that the nearest neighbor of the eBG54 Agona cluster was a strain of the extremely rare serovar Soerenga. The MLST database only includes two Soerenga strains, one of which is in MLST ST1659 (Fig 1) as is the isolate of Timme *et al*. ST1659 shares 0/7 alleles with any of the STs in eBG54, strongly indicating that they are not closely related. According to the phylogeny of Timme *et al*. (http://treebase.org/treebase-web/search/study/summary.html?id=14912), the split between eBG54 and Soerenga is an ancient event, which occurred at 62% of the TMRCA of all *S*. *enterica* subspecies *enterica*. However, we are concerned that the apparent clustering of Agona and Soerenga is an artefact because the tree topology and branch lengths are inaccurate near the root of subspecies *enterica* (Technical Appendix 2). Timme *et al*. calculated SNPs identified by the 95% 25 kmer approach of kSNP2, which identifies SNPs whose flanking 12 bp segments are identical in at least 95% of the genomes. These calculations do not account for recombination, and kSNP2 yields inaccurate topologies for bacteria of the frequent recombination and high genetic diversity of subspecies *enterica* [16]. We are also skeptical about the accuracy of branch lengths calculated by Timme *et al*. because they did not implement measures to remove clustered SNPs or homoplasies in repetitive or recombinant regions. The only filtering that was applied was to use the kmer approach, which removes highly divergent regions but does not address all clustered SNPs or homoplasies. Note that the number of unique (kmer) SNPs on any of the branches indicated by Timme *et al*. [14] is only a very low fraction of all SNPs, indicating that the vast majority of SNPs in the phylogeny are homoplastic, and the branch lengths are probably highly inaccurate. For example, the branch length (0.04) from the root of subspecies *enterica* to the split between eBG54 and Soerenga should include thousands of mutations (≈ 0.04 * 119,750), but those branches are annotated with only 29 unique SNPs. These combined issues raise questions about both the branching order and the branch lengths in the tree of Timme *et al*. Even if taken at face value, this tree does not show a close relationship between ST1659 (Soerenga) and eBG54 (Agona).

## Critique of Data and Analyses Presented in Pettengill [15]

### 1. Underpowered and flawed analysis of data

#### Pettengill writes

‘For simplicity, I ran BEAST analyses including only four samples (Table 1) from the original publication of Zhou et al. …, which were arbitrarily chosen to capture the evolutionary breadth contained in that study. I also ran BEAST including the closest known serovar to Agona, S. Soerenga, which was identified based on a large phylogeny including 76 S. *enterica* ssp. *enterica* serovars … Given that the SNP matrix within Zhou et al. … was not available, I downloaded the assemblies and performed a whole genome sequence alignment using Mugsy v.1.2.3 with default settings… The program ClonalFrameML v1.25 … was then used to detect recombination, which can bias estimates of TMRCA and other evolutionary dates.’

**Table 1:** Age estimates and Bayes Factors from BEAST analyses of 864 non-repetitive, non-recombinant, non-homoplastic core SNPs from 73 eBG54 (Agona) genomes.

Clock:	Relaxed Clock				Strict Clock	
Model:	GMRF		Constant Population Size		Constant Population Size	
	Mean MRCA	95% Confidence Interval	Mean MRCA	95% Confidence Interval	Mean MRCA	95% Confidence Interval
**2013:**						
**HME**	-5860227		***-5860225***		-5860298	
**Basal node**	1932	1918–1945	1799	1618–1928	1839	1765–1894
**rootHeight**	1932	1917–1944	1800	1620–1927	1838	1764–1893
**2015:**						
**HME**	-5860231		***-5860229***		-5860300	
**Path sampling**	***-5860609***		-5860636		-5860702	
**Stepping-stone**	***-5860599***		-5860606		-5860665	
**Basal node**	1931	1915–1944	1803	1635–1919	1839	1767–1894
**rootHeight**	1931	1916–1944	1805	1636–1920	1839	1767–1894

Note: Highest Bayes factors are indicated by bold, italic fonts. Path sampling and Stepping-stone analyses were performed along a series of 100 steps along the path, with a chain of 1M samples per step.

**Fig 2:**
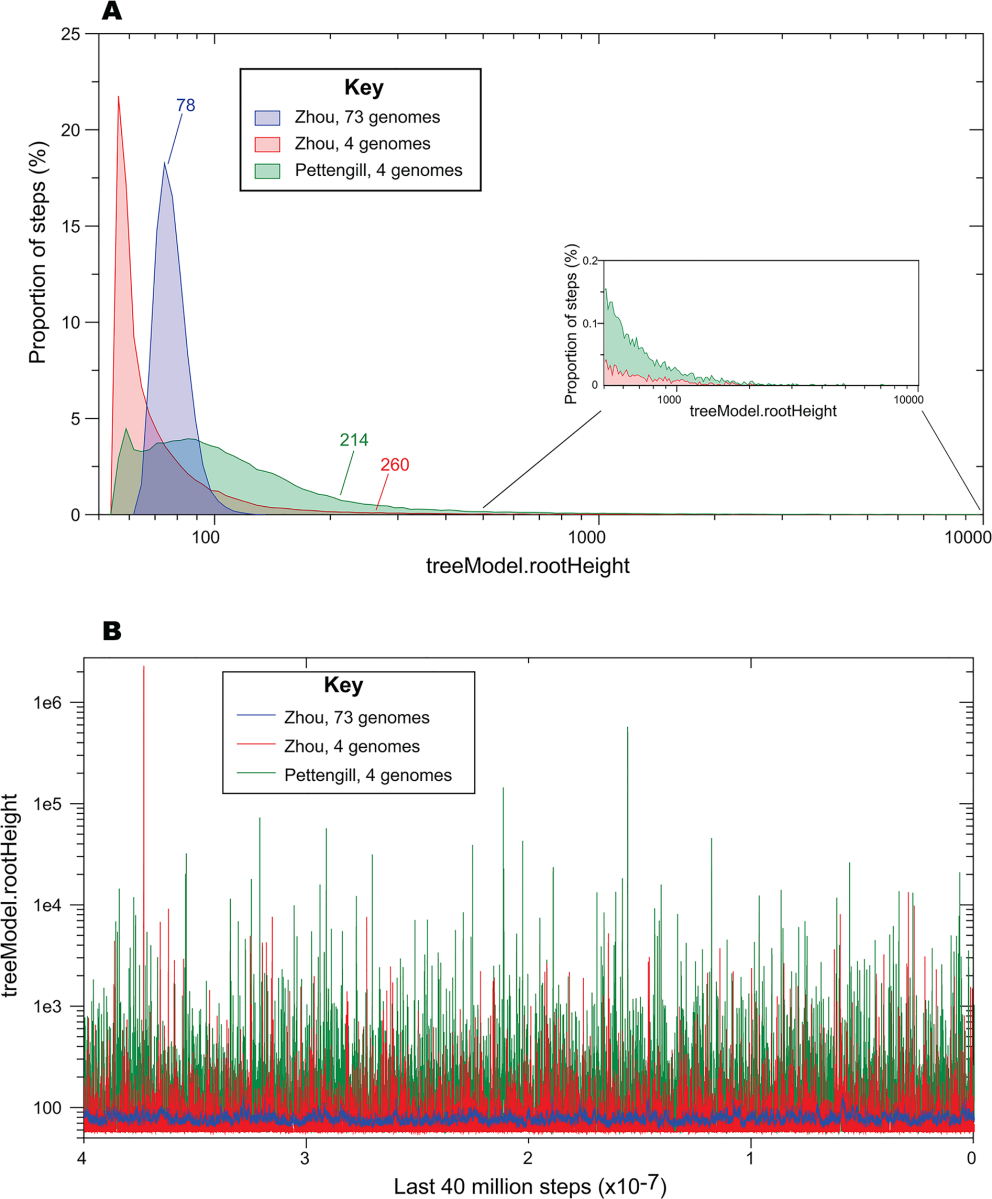
Comparisons of treeModel.rootHeight estimates by BEAST with different SNP calls and different numbers of genomes. A. Distribution of numbers of estimates of rootHeight as a percentage of all estimates in BEAST analyses according to the best model in Table 1. The numbers were from samples taken every 1000 steps over a total of 200 million steps (4 genomes) or 50 million steps (73 genomes), after excluding the first 10 million steps as burn-in. Mean values of rootHeight are indicated next to arrows. Inset, different scale for values of rootHeight over 500 years. B. Representation of the individual rootHeight values for each sample over the last 40 million steps. Pettengill, 4 genomes: uses the SNP calls calculated by Pettingill [15]; Zhou, 4 genomes: uses the SNP calls for the same four genomes extracted from the core genomes in Zhou *et al*., [9]; Zhou 73 genomes, uses the core genome SNPs from all 73 genomes in [9].

There are multiple inaccuracies and problems with these statements. Firstly, the use of only four samples underestimates the fluctuations of population size and mutation rate within Agona, and results in dramatic problems with dating estimates (Fig 2, Technical Appendix 3). Four samples are also too few for accurate detection of recombination with ClonalFrameML. Secondly, filtering was not performed for repetitive or mobile DNA, both of which can lead to incorrect SNP calls due to nonspecific alignments with paralogous genes. The SNPs called with this approach were much less appropriate for dating than those used by Zhou *et al*. (Fig 2, Technical Appendix 3). What is equally disturbing is that the original SNP calls of Zhou *et al*. were never examined, although those SNP calls reflected state of the art detection of non-homoplastic mutations from 73 genomes after filtering of recombinant, repetitive and mobile DNA. Pettengill notes that the SNP matrix was not available, and we have now uploaded the SNP matrix for the convenience of other users (http://figshare.com/articles/SNP_matrix_for_73_Agona_genomes/1434661). However, the mutational SNPs were already listed in Supplementary Dataset 4 by node position in the tree in Fig 4 of Zhou *et al*., which is more informative than a simple SNP matrix. It would have been readily possible to recover the tree from Fig 4 using TreeSnatcherPlus [17] and then generate a SNP matrix by applying each mutation in Supplementary Dataset 4 to all genomes descended from the corresponding branch. Alternatively, the SNP matrix could have been obtained from the authors by sending an email. Instead, Pettengill chose to perform an underpowered, flawed *de novo* analysis of only four genomes.

### 2. Inappropriate claims for age estimations

#### He also writes

‘I analyzed the four Agona samples contained in Zhou et al. … under the best fitting model described in the paper (e.g., uncorrelated lognormal clock rate and Gaussian Markov random fields (GMRF) tree model that allows for historical fluctuations in population size). Under this analysis, the age of the most basal node of the Agona isolates sampled was 1927 or 88 ybp (years before present) (CI95% 57–512 ybp) (Fig 2A), which is quite similar on an evolutionary scale to the year 1932 that was observed in Zhou et al.… However, the mean estimate of the actual TMRCA (treeModel.rootHeight from the BEAST output) was nearly three times as old (313 ybp; 95%CI: 57–295 ybp) and illustrates the difference that exists between an estimate of the age of the most basal node in a phylogeny and an estimate of the time at which alleles segregating in the dataset coalesce back to a single common ancestor (i.e., the TMRCA). Had Zhou et al. … correctly identified the TMRCA their incorrect estimate of the date of emergence of Agona would have likely been hundreds of years older than what they reported, which was based on the age of the most basal node.’

The most striking problem with these statements is that treeModel.rootHeight was nearly three times as old as the age of the basal node. According to our experience, rootHeight and age of the basal node are normally nearly identical when the Bayesian runs have converged. A personal communication from O. Pybus, one of the lead authors of the BEAST software, indicates that they are identical by definition. To demonstrate this, we reproduce the dating estimates according to both rootHeight and basal node that were measured by Zhou *et al*. [9] (Table 1). At that time, the primary tool for calculating Bayes Factors to compare the likelihood of the three models was HME (harmonic mean estimator) which yielded very minor differences between the relaxed clock models invoking variable (GMRF) and constant population sizes, both of which were much preferred over a strict clock model. We chose the GMRF model for discussion in the publication because we anticipated that major increases in population size would have resulted from the geographic expansions in the 1960’s of Agona from South America to the rest of the world. Since 2013, newer methods based on Path sampling and Stepping-stone sampling models have been implemented that are more reliable than HME [18]. We have therefore recalculated the BEAST analyses and applied these criteria to identify the best model. Both Path Sampling and Stepping-stone sampling (Table 1) indicated that the model favored by Zhou *et al*., a relaxed clock with GMRF, is much preferred to one with a constant population size, and both relaxed clock models are much preferred to a strict clock.

The data in Table 1 also show that the estimates of age and their 95% confidence limits were almost identical between the basal node and treeModel.rootHeight for all models and all comparisons, negating his claim that we used the wrong BEAST parameters to estimate age as well as his claim that the TMRCA predates the basal node. Furthermore, Pettengill’s discrepancy of threefold is a convincing indicator of problems with his analyses, which we confirmed by independent BEAST analyses of the SNPs he chose versus those identified by Zhou *et al*. (Fig 2, Technical Appendix 3). The results in Fig 2 also show that all his date estimates are uncertain.

### 3. Age of eBG54 (Agona)

Pettengill also criticizes the conclusion in the Abstract by Zhou *et al*. that ‘only 846 single nucleotide polymorphisms (SNPs) have accumulated in the non-repetitive, core genome since Agona evolved in 1932.’ In the text, Zhou *et al*. also wrote ‘A more sophisticated Bayesian analysis (BEAST) indicated that the MRCA evolved in or before 1932 (CI95%: 1918–1945) (Table S4, Fig 4A).’, ‘An alternative relaxed clock model with constant population size yielded a slightly better fit (higher Bayes factor) than the GMRF model, and a date for the MRCA of 1799 (CI95%: 1618–1928) (Table S4).’, and ‘These calculations indicate that Agona is a recently evolved pathogen, which likely arose about 80 years ago. Consistent with this interpretation, Agona was first identified in 1952.‘

In retrospect, it would probably have been better had the Abstract contained the words ‘in or before 1932’, but the text is quite clear about the broad range of confidence intervals for the age of MRCA. The text also clearly implies that the MRCA is simply the coalescent of current diversity, whose genetic composition may not have differed greatly from an ancestor which existed earlier, but did not leave descendants that have survived to current times of sampling, or whose descendants were not included in the sample.

### 4. Problems with calculating divergence time

Pettengill also demands that the age of divergence of eBG54 from the nearest outgroup should have been calculated in order to estimate the date of emergence of eBG54, and he attempts to estimate an upper bound on the date of emergence by calculating the divergence time from ST1659 (Soerenga). We dispute that using an arbitrary, only distantly related outgroup results in a more accurate estimation of emergence time than is encompassed by the confidence limits of the TMRCA for eBG54, or for most of the other eBGs that are currently known in subspecies *enterica*. Firstly, our arguments in the Introduction show that close relatives to eBG54 have not yet been identified, so all estimates of divergence time are vast overestimates. Secondly, it remains to be demonstrated that divergence times near the root of the *enterica* tree can be accurately estimated with current data and methodologies. In particular, we are very skeptical about the abilities of any modern algorithms, including ClonalFrameML, to accurately identify mutational changes near the root rather than recombinational changes, which are not necessarily acquired according to the same clock rates. Thirdly, it is not advisable to extrapolate mutation rates over a timeframe of many millennia that were calculated from a sample taken during 70 years, because short term clock rates tend to be faster than long term clock rates [19,20]. Genomic sequences of ancient DNA from subspecies *enterica* that existed millennia ago would be needed to calibrate such estimates. Finally, we feel that no analysis of four eBG54 genomes plus one ST1659 genome could provide accurate estimates of the MRCA of the ingroup as well as the divergence time between both lineages, and that any attempt to do so should have taken account of the different approaches needed for intra-clade coalescents *versus* inter-species divergences [21,22].

## Conclusions

We reject the critique by Pettengill as being unfounded and/or not capable of being currently implemented. We also continue to claim that eBG54 derives from a common ancestor which existed in or before 1932.

## Technical Appendix

### 1. Inappropriate simulations

Pettengill [15] performs simulations of the coalescent process for a sample of ten individuals from one population and one individual from a second population (outgroup). One set of simulations was performed assuming a deep divergence time, in which case the sequences from the first population coalesced much more recently with each other than with the outgroup. A second set of simulations was also performed assuming a much more recent divergence time, in which case intra-population and inter-population coalescent times overlapped. These observations are explained as reflecting the time needed for complete lineage sorting, and used to justify the concept that including an outgroup is necessary to reliably calculate divergence times within a population of interest. Although correct from a theoretical population genetic perspective, these analyses and conclusions are inappropriate for dating Agona.

Pettengill used the program ms in its default mode, which performs simulations assuming a constant population size, and used an island model (parameter–I) of complete spatial separation between the two populations. Instead, most recent estimates of the ages of bacterial pathogens have needed to invoke dramatic changes in population size [8,23,24]. In addition, many genetically monomorphic bacterial populations, such as serovar Agona, are both clonal and undergo epidemic spread. These populations can undergo very dramatic, repeated bottlenecks, which reduce the effective population size to only a single cell [25], and frequently result in death of lineages, aspects that are not adequately accounted for by classical population genetic algorithms [23,26], including the simulations performed by Pettengill.

### 2. Estimation of the accuracy of the topology of Timme *et al*. [14]

According to Hall [16], topologies based on kSNP2 become inaccurate when recombination rates and genetic diversity are high. Didelot et al. [5] calculated that the average frequency of recombination events per nucleotide substitution (ρ/θ) in subspecies *enterica* ratio was 0.37 (CI95% 0.33–0.41). In order to estimate genetic diversity within the dataset of Timme *et al*., we aligned all 156 genomes from that analysis against a reference genome (Choleraesuis str. SC-B67). For consistency with the kSNP analysis, we did not filter repetitive regions or mobile elements. A total of 561,132 SNPs were identified in 3,897,271 bps that are present in at least 95% of genomes, which equals a genetic diversity of 14.4%. According to the simulation by Hall, the topology of >50% of the branches inferred by kSNP2 is expected to be inaccurate for these values of diversity and recombination rate. We would anticipate that the tips of the tree, which have the strongest signals are least likely to be inaccurate whereas branches near the root are most likely to be wrong.

### 3. Accuracy of rootHeight versus SNP calling and numbers of genomes

It seemed intuitive to us that calling SNPs without excluding repetitive and/or homoplastic DNA would cause problems with genealogies and dating. It also seemed intuitive that the analysis of only four genomes would be less accurate than an analysis of 73. Finally, we did not expect ClonalFrameML to be as accurate in identifying recombinant segments with only four genomes as it would be with 73. However, we were unable to identify a citation which strongly supported these intuitions. We have therefore reanalyzed the SNPs from four genomes from Pettengill [15] who had attempted to remove recombinant regions with ClonalFrame ML but did not exclude repetitive/homoplastic SNPs. These SNPs were subjected to BEAST analyses using the relaxed clock GMRF model, which has the highest Bayes factors in Table 1 (Fig 2), with similar results between two independent runs,. We also performed two runs on the same four genomes, but using the SNPs in the core genome according to Zhou *et al*., in which recombinant/homoplastic segments had been removed after comparisons of all 73 genomes, and which also excluded repetitive DNA. The results showed that treeMode.rootHeight is extremely heterogeneous with the SNPs called by Pettengill, and forms a bimodal distribution (Fig 2). SNPs called by Zhou *et al*. yielded a tighter, monomodal distribution of rootHeight. However, in both datasets, the distributions of rootHeight are highly asymmetrical, with a very dramatic tail extending up to ~1 million years. This long tail also resulted in estimated mean rootHeights of >200 years, which is much greater than the single peak found with the Zhou *et al*. data or the two peaks found for Pettengill’s SNPs. In contrast, the original analysis of 73 genomes yielded a very tight, symmetrical distribution, did not include any values greater than 130 years, and estimated mean rootHeight as 78 years, which was very similar to the peak value. These observations were not due to lack of convergence because the effective sample size (ESS) was high for all analyses (four genomes: >5,000; 73 genomes: >400). Instead, they cast grave doubts on the validity of Pettengill’s approach to dating the age of Agona, and indicate that dating should be based on non-recombinant, non-repetitive core SNPs from larger numbers of genomes.
